# Distinct Impacts of Prenatal and Postnatal Phthalate Exposure on Behavioral and Emotional Development in Children Aged 1.5 to 3 Years

**DOI:** 10.3390/toxics12110795

**Published:** 2024-10-31

**Authors:** Yi-Siang Huang, Pi-Lien Hung, Liang-Jen Wang, Chih-Min Tsai, Chang-Ku Tsai, Mao-Meng Tiao, Hong-Ren Yu

**Affiliations:** 1 Department of Pediatrics, Kaohsiung Chang Gung Memorial Hospital, Kaohsiung 833, Taiwan; hiss2223@gmail.com (Y.-S.H.);; 2 Department of Child and Adolescent Psychiatry, Kaohsiung Chang Gung Memorial Hospital, Kaohsiung 833, Taiwan; 3 College of Medicine, Chang Gung University, Taoyuan 330, Taiwan; 4 Institute for Translational Research in Biomedicine, Chang Gung Memorial Hospital, Kaohsiung 833, Taiwan

**Keywords:** prenatal, postnatal, phthalate, toddlers, child behavior checklist, behavioral development, urine sample

## Abstract

Development is a continuous process, but few studies have assessed the simultaneous impact of prenatal and postnatal phthalate exposure on children’s behavioral and emotional development. A total of 491 mother–child pairs from the general population in southern Taiwan were studied from 2021 to 2022. Urinary concentrations of bisphenol A (BPA) and phthalate metabolites—mono-ethyl phthalate (MEP), mono-n-butyl phthalate (MnBP), mono-benzyl phthalate (MBzP), and mono-2-ethylhexyl phthalate (MEHP)—were measured in pregnant mothers during the second trimester and in their corresponding children aged 1.5 to 3 years. Behavioral symptoms in children were evaluated using the Child Behavior Checklist (CBCL). Odds ratios (ORs) represent a 1-unit increase in log10-transformed creatinine-corrected maternal urine concentrations. Prenatal maternal urinary MnBP levels were associated with total problems (OR = 19.32, 95% CI: 1.80–43.13, *p* = 0.04), anxiety (OR = 33.58, 95% CI: 2.16–521.18, *p* = 0.01), and sleep problems (OR = 41.34, 95% CI: 1.04–1632.84, *p* = 0.04) in children. Additionally, urinary MnBP levels in children correlated with total problems (OR = 7.06, 95% CI: 1.01–49.05, *p* = 0.04) and internalizing problems (OR = 11.04, 95% CI: 1.27–95.72, *p* = 0.01). These findings suggest that prenatal and postnatal exposure to dibutyl phthalate (DBP), metabolized as MnBP, distinctly affects children’s behavioral development.

## 1. Introduction

Endocrine-disrupting chemicals (EDCs) interfere with the endocrine system by mimicking natural hormones, such as those produced by the pituitary, thyroid, and adrenal glands [1]. EDCs can alter the metabolism of endogenous hormones, interfere with both genomic and non-genomic pathways, disrupt feedback regulation mechanisms, and affect neuroendocrine cell function. In some cases, they may cause changes in DNA methylation or histone modification, resulting in genomic instability [2]. Among the many EDCs, bisphenol A (BPA) and phthalates have aroused particular concern. Due to the mass production of plastic products, BPA and phthalates are now ubiquitous in everyday life [3,4]. BPA is widely used in food and beverage packaging, medical devices, thermal paper, and dental products [5]. The application range of phthalates depends on their molecular weight and chemical properties: low molecular weight (LMW) phthalates are often found in cosmetics, lotions, aerosols, and coatings of certain medications [6,7], while high molecular weight (HMW) phthalates are primarily used as plasticizers and adhesives in products such as food containers, flooring, wall coverings, and medical tubing [8].

Human brain development begins early in pregnancy, with rapid growth occurring in mid-gestation. By the end of the embryonic stage, the foundational structures of the brain and central nervous system have formed [9]. The majority of cortical neurons are formed from early fetal development through mid-gestation. Throughout pregnancy, the brain undergoes significant morphological changes, leading to the formation of the brain’s gyri and sulci by late gestation [9,10]. Fetal programming refers to the concept that environmental factors during critical or sensitive periods of fetal and early childhood have lasting impacts on an individual’s health by altering metabolic and physiological systems [11]. The first two years of life are crucial for neurodevelopment, significantly influencing cognitive, emotional, language, and social skills [12]. During this period, synaptic connections between neurons rapidly increase, forming intricate neural networks. This neuroplasticity allows the child’s brain to adapt to its environment and acquire new skills [12]. However, adverse events during this period may induce epigenetic changes, which can affect long-term health and disease susceptibility [13].

Recent research has identified associations between bisphenol A (BPA), phthalates, and a range of health disorders. BPA has been linked to health conditions such as adult-onset diabetes, reduced semen quality, and polycystic ovary syndrome, while phthalates have been correlated with preterm birth, reduced anogenital distance in boys, childhood obesity, and impaired glucose tolerance [14]. BPA and phthalates are known to notably impair the vasculature, structure, and function of the placenta [15]. It is also well established that certain phthalate metabolites can cross the placental barrier, entering fetal circulation and influencing the rapidly developing fetal brain. Animal studies have demonstrated that perinatal exposure to di-(2-ethylhexyl) phthalate (DEHP) is linked to anxiety- and depression-like behaviors in rats [16]. As more human studies indicate that prenatal exposure to phthalates have adverse effects on children’s neurodevelopment, research in this area is receiving increasing attention [6,8].

Although previous studies have explored the association between prenatal phthalate exposure and children’s behavioral development, the results remain inconsistent [17,18,19,20]. Therefore, this study collected the concentrations of phthalate metabolites in the urine of pregnant women and young children, as well as assessments of children’s neurodevelopment, aiming to investigate the effects of prenatal and postnatal phthalate exposure on children’s neurobehavioral development.

## 2. Materials and Methods

### 2.1. Study Population

This study employed a prospective birth cohort design to investigate the effects of prenatal and postnatal exposure to phthalates and BPA on early childhood behavioral development. Pregnant women attending the Obstetrics and Gynecology Clinic at Kaohsiung Chang Gung Memorial Hospital were recruited between July 2021 and December 2022, with data collection starting upon receiving informed consent. Basic demographic and physical characteristics were collected during the second trimester of the pregnancy. At birth, physiological data were recorded, and at 1.5 to 3 years of age, the children underwent behavioral assessments using the Child Behavior Checklist for ages 1.5–5 (CBCL/1.5–5). Preterm infants were excluded to minimize potential confounding factors.

### 2.2. Cognitive and Behavioral Assessments for Children

Cognitive and behavioral assessments were conducted using the CBCL/1.5–5, a caregiver-reported questionnaire that evaluates a broad spectrum of emotional and behavioral issues in children. The CBCL has been validated for use in research and clinical settings [18]. Specifically tailored for preschool-aged children, the CBCL/1.5–5 includes 100 items and was completed by the child’s primary caregiver, most often the mother in this study. Each item is assessed reflecting the child’s behavior during the previous two months, with scores given as 0 (not true), 1 (somewhat true), or 2 (very true) [21]. T-scores are used to interpret the results, with scores of 70 or higher classified as “clinical range”, scores between 65 and 70 as “borderline range”, and scores below 65 as “normal range”. The CBCL also includes syndrome scales for internalizing problems (covering emotional reactivity, anxiety/depression, somatic complaints, and withdrawal), externalizing problems (such as attention issues and aggressive behavior), and sleep problem syndromes. DSM-oriented scales are also available. For the total problem score, a score below 60 is considered normal, 60–63 is borderline, and scores above 63 are clinical [22]. The CBCL/1.5–5 has been demonstrated to possess strong reliability and validity. All participants provided informed consent as approved by the Institutional Review Board (IRB 202100426A3) of Chang Gung Memorial Hospital.

### 2.3. Prenatal Phthalate Collection

Phthalate metabolites have been detected in various biological fluids, such as urine, serum, semen, breast milk, and saliva. Since phthalates can cross the placental barrier, they have also been detected in amniotic fluid. However, urine samples typically exhibit higher metabolite concentrations and are easier to collect [23]. Urine samples were analyzed to measure various phthalate metabolites, such as monobenzyl phthalate (MBzP), monoethyl phthalate (MEP), mono-n-butyl phthalate (MnBP), mono(2-ethylhexyl) phthalate (MEHP), as well as bisphenol A (BPA). These measurements were performed using liquid chromatography coupled with electrospray ionization tandem mass spectrometry. Metabolite quantification was performed using an internal isotope standard curve method, based on a modified version of the protocol developed by Blount et al. [24]. In summary, each urine sample was spiked with 50 μL of an internal standard solution containing 200 ng/mL of 13C12-labeled phthalate metabolites, along with 1 mL of 2 M ammonium acetate buffer (prepared by dissolving 1.54 g of ammonium acetate in 10 mL of HPLC-grade water) and 20 μL of β-glucuronidase. Samples were then incubated at 37 °C for one hour, followed by two rounds of extraction with 4 mL of ethyl acetate. The samples were gently mixed, and after centrifugation at 4000 rpm for 15 min, the organic layer was separated from the fat-containing non-polar layer. Chromatographic separation was carried out using a Shim-pack GIST C18 column (2.1 mm × 100 mm, 2 μm; Shimadzu, Japan). The analysis of the metabolites was conducted on a Shimadzu 8050 Triple Quad liquid chromatograph mass spectrometer, using a Shimadzu LC-30 series HPLC system. Detection limits were 1 μg/μL for MEP and BPA, and 0.8 μg/μL for MnBP, MBzP, and MEHP [25]. To minimize the interference of urine concentration, urinary phthalate metabolites were corrected using urinary creatinine. Urinary creatinine levels were measured using spectrophotometry to normalize the metabolite concentrations [26,27].

### 2.4. Statistical Analyses

Several factors regarding the mother, child, and family, including social determinants, were recorded. Maternal characteristics included age at enrollment, household size, parental education levels, smoking and alcohol use during pregnancy, and pre-pregnancy BMI. Statistical analyses were performed using IBM SPSS Statistics 25.0, and independent *t*-tests were conducted to compare demographic differences between boys and girls. Due to the right-skewed distribution of phthalate metabolites, a log10 transformation was applied to align the data with model assumptions. T-scores for CBCL symptoms followed a roughly normal distribution. CBCL scores were analyzed as continuous variables or as categorical variables to classify children into normal, borderline, or clinical ranges. Multinomial logistic regression was used to estimate odds ratios (ORs) comparing children in the clinical range with those in the normal and borderline ranges. The model also assessed the likelihood of clinical behavioral problems (above the 95th percentile) compared to scores below the 90th percentile, adjusting for sex [28,29], parents’ age [30], parents’ smoking status [31], maternal parity [32], parents’ education level [33], and mother’s BMI [34]. For continuous outcomes, multivariate linear regression was used to assess the relationship between prenatal or postnatal urinary phthalate levels and children’s behavior problem scores. Independent *t*-tests were used to examine the differences in CBCL scores between boys and girls, and to compare prenatal and postnatal urinary phthalate metabolite concentrations by sex. Paired *t*-tests were used to evaluate differences between the phthalate metabolite concentrations of mothers and their paired children. Pearson correlation analysis was performed to assess the correlation between prenatal and postnatal phthalate metabolite levels.

We conducted additional sensitivity analyses to assess the robustness of our results. False discovery rate (FDR) correction was applied to the comparison of phthalate metabolite levels between mothers (prenatal) and infants (postnatal) using the Benjamini–Hochberg method to adjust the *p*-values. Additionally, since the other results utilized linear regression models and logistic regression models, we conducted a stratified analysis for the subgroup of mothers with an education level above high school and those with a parity of one, in order to enhance the robustness of our findings.

## 3. Results

### 3.1. Descriptive Statistics for the Enrolled Population

Data were collected from 491 mother–child pairs, consisting of 245 boys and 246 girls. The average age of the children was 40.96 months (range: 23–73 months), with boys averaging 41.13 ± 7.36 months and girls averaging 40.78 ± 8.05 months. The mean maternal age was 33.28 ± 4.43 years (range: 21–43 years). Of the children, 62.5% were firstborns. The majority of the parents had a university-level education. There were no significant differences between the boys and girls regarding parental age, education level, or smoking habits. The baseline demographic information is shown in Table 1.

Table 2 presents the CBCL scores for various subscales. Overall, the mean T-score for total problems was 52.05. The mean scores were 54.33 for internalizing problems and 47.75 for externalizing problems. Among the CBCL subscales, boys scored higher than girls on the attention deficit hyperactivity disorder (ADHD) problem subscale. No significant differences were observed between boys and girls in any of the other CBCL subscales.

The distribution of children across the ‘normal’, ‘borderline’, and ‘clinical’ ranges is detailed in Supplementary Table S1. Among all participants, 13.6% were in the clinical range for total problems on the broadband scales. Additionally, 19.1% were in the clinical range for internalizing problems, and 4.1% for externalizing problems. The three most frequently reported syndromes in the clinical range were internalizing problems (19.1%), affective problems (14.3%), and somatic complaints (13.8%). When analyzed by sex, the most frequently reported syndromes in boys were internalizing problems (20.0%), somatic complaints (13.9%), total problems (13.5%), and affective problems (13.5%). For girls, the most commonly reported syndromes were internalizing problems (18.3%), affective problems (15.0%), somatic complaints (13.8%), and total problems (13.8%).

### 3.2. Maternal Urinary MEP and MnBP Levels Are Higher than Their Young Children

**Figure 1:**
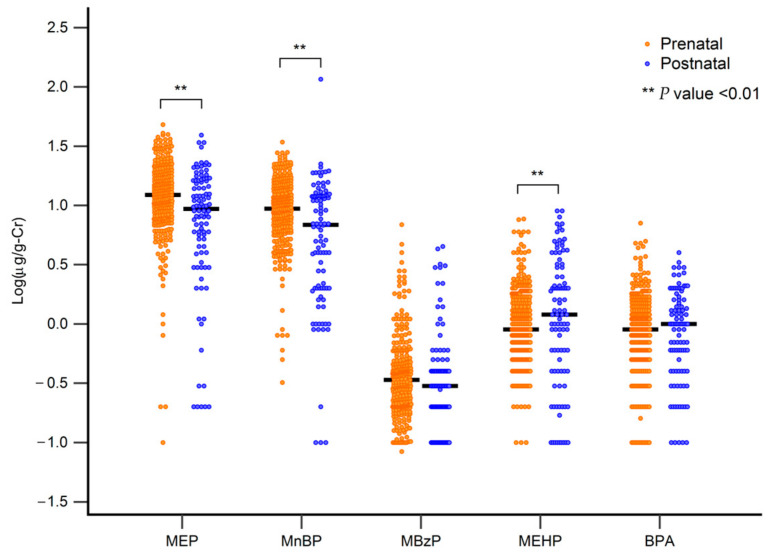
Differences in mother (prenatal) and infant (postnatal) metabolite levels. ** *p* < 0.01. (MEP = Mono-ethyl phthalate, MnBP = Mono-n-butyl phthalate, MBzP = Mono-benzyl phthalate, MEHP = Mono-2-ethylhexyl phthalate, BPA = Bisphenol A).

Table 3 displays the concentrations of phthalate metabolites in maternal and child urine samples collected prenatally and postnatally. We measured four phthalate metabolites and BPA from both maternal and child urine samples. The detection rate for relevant phthalate metabolites and BPA in this study was 100%. In mothers, the highest metabolite concentration was MEP (range: 0.1–48, mean = 14.07), and the lowest was MBzP (range: 0.084–6.9). In children, the highest concentration was also MEP (range: 0.2–39.2, mean = 10.72), and the lowest was MBzP (range: 0.1–4.5). No differences were found in maternal phthalate metabolite concentrations between mothers of male and female children; however, boys had higher concentrations of MBzP in their urine compared to girls. Next, we compared phthalate metabolite concentrations between mothers and children. Mothers had higher concentrations of MEP and MnBP than their children, while MEHP concentrations were higher in children than in mothers (Figure 1). Additionally, we compared the concentrations of phthalate metabolites in the urine of pregnant women worldwide. We observed differences in measurement units when compared to other international studies. In comparison with studies conducted in Taiwan, our results showed lower urinary phthalate concentrations. This difference may be related to the study period and the specific stages of pregnancy during which the data were collected (Supplementary Table S2).

**Figure 2:**
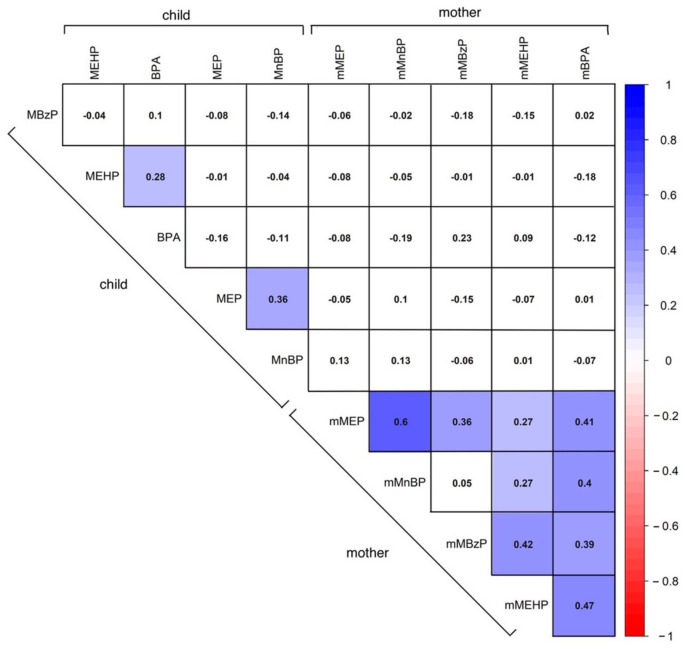
Correlation heatmap between various chemical concentrations in paired children and mothers. The color gradient from blue to red indicates the strength and direction of the correlations, with blue representing positive correlations and red representing negative correlations. Notable correlations include a positive correlation of mothers’ metabolites of urine plasticizers and a positive correlation between child MnBP and MEP (*r* = 0.36) and MEHP and BPA (*r* = 0.28). There was no statistical significance between children and mothers. (MEP = Mono-ethyl phthalate, MnBP = Mono-n-butyl phthalate, MBzP = Mono-benzyl phthalate, MEHP = Mono-2-ethylhexyl phthalate, BPA = Bisphenol A).

To explore whether the differences in phthalate metabolite concentrations between mothers and children remained significant in paired analyses, we conducted a paired comparison of cases with both prenatal and postnatal data. We found that, apart from MEP and MnBP being higher in mothers, there were no significant differences in MEHP levels between mothers and their paired children (Supplementary Figure S1). We also examined the correlation between maternal and child phthalate metabolite concentrations in paired samples from the same environment. The results showed a strong positive correlation between various maternal phthalate metabolites. However, no correlation was observed between the concentrations of phthalate metabolites in mothers and those in their corresponding children. We found weakly positive correlations between child concentrations of phthalate metabolites levels of MEHP and BPA, as well as between MEP and MnBP (Figure 2).

### 3.3. Prenatal Maternal Urinary MnBP Level Was Associated with Anxiety, Sleep Problems, and Total Problems in Children Aged 1.5- to 3-Years-Old

To assess whether prenatal exposure to phthalates was correlated with children’s emotional and behavioral problems, while controlling for potential confounding factors, multinomial regression analyses were conducted. We conducted regression analyses to examine the association between maternal urinary phthalate metabolite concentrations and CBCL T-scores (Table 4).

The coefficients represent T score elevations associated with a 1-unit increase in log10-transformed creatinine-corrected concentrations of maternal urinary phthalate metabolite. We found that prenatal maternal MEP exposure was associated with CBCL T-scores for pervasive developmental problems (PDP) (coefficient = 0.12, CI: 0.24–0.81, *p* = 0.03) and ADHD (coefficient = 0.13, CI: 0.37–4.00, *p* = 0.01). Prenatal maternal urinary MnBP concentration was associated with anxious/depressed (coefficient = 0.12, CI: 0.08–4.44, *p* = 0.04), aggressive behavior (coefficient = 0.12, CI: 0.67–4.61, *p* = 0.04), PDP (coefficient = 0.14, CI: 0.71–6.88, *p* = 0.01), ADHD (coefficient = 0.18, CI: 1.20–5.21, *p* = 0.01), and oppositional defiant disorder (ODD) (coefficient = 0.16, CI: 0.63–4.06, *p* = 0.01). Prenatal maternal urinary MBzP concentration was associated with somatic complaints (coefficient = −0.15, CI: −6.99 to −0.97, *p* = 0.01) and aggressive behavior (coefficient = 0.12, CI: 0.19–3.86, *p* = 0.03). Thereafter, we evaluated the association between maternal phthalate metabolite concentrations and CBCL scores of children in the clinical range, both unadjusted (Supplementary Table S7) and adjusted for sex, parental age, smoking status, education level, maternal parity, and maternal BMI (Table 5). ORs represent a 1-unit increase on log10-transformed creatinine-corrected maternal urine concentrations. After adjusting for possible confounding factors, we found that prenatal maternal urinary MnBP level was associated with anxiety (OR = 33.58, 95% CI: 2.16–521.18, *p* = 0.01), sleep problems (OR = 41.34, 95% CI: 1.04–1632.84, *p* = 0.04), and total problems (OR = 19.32, 95% CI: 1.80–43.129, *p* = 0.04).

### 3.4. Children’s Urinary MnBP Level Was Associated with Total Problems and Internalizing Problems in Children Aged 1.5- to 3-Years-Old

The relationship between postanal phthalates exposure and children behavior development was investigated. We also conducted regression analyses to examine the association between children’s urinary phthalate metabolite concentrations and CBCL T-scores (Table 6). The phthalate metabolite concentrations of children and CBCL scores within the clinical range was analyzed, examining both unadjusted and adjusted models for factors such as sex, parental age, smoking status, education level, maternal parity, and maternal BMI (Supplementary Table S8) (Table 7). The urinary MnBP concentration of children was associated with total problems (OR = 7.06, 95% CI: 1.01–49.05, *p* = 0.04) and internalizing problems (OR = 11.04, 95% CI: 1.27–95.72, *p* = 0.01). Children’s urinary MBzP level was associated with externalizing problems (OR = 0.01, 95% CI: 0.01–0.46, *p* = 0.02), and the MEHP level was associated with externalizing problems (OR = 0.10, 95% CI: 0.01–0.94, *p* = 0.04). However, the sample size was too small to distinguish between groups with CBCL scores that reached clinical significance for attention problems.

### 3.5. Sensitivity Analysis

We conducted stratified analyses to assess the robustness of our main findings. Specifically, we divided the data based on certain key maternal and infant characteristics to verify whether the results remained consistent across subgroups. (Supplementary Tables S3–S6) These subgroups included mothers with an education level of high school or above, as well as those with a parity of one. In each case, the stratified analysis indicated that the association between urinary concentrations of MBzP and MEHP in mothers or infants and CBCL scores showed some variation. However, the main results regarding MnBP remained unchanged. The stability of our findings across different conditions supports the robustness of our analytical approach, adding confidence to the validity of our study.

## 4. Discussion

Children’s emotions and behaviors serve as crucial mediators of the interaction between the individual and the environment, influencing aspects such as adaptability, interpersonal relationships, and academic performance [35]. Scholars from the evidence-based approach categorize children’s emotional and behavioral problems into two dimensions: internalizing behaviors and externalizing behaviors. Internalizing problems refer to internal conflicts or distress exhibited by children, often manifested as depression, anxiety, or withdrawal behaviors. In contrast, externalizing problems arise from conflicts between the individual and the environment, resulting in responses that affect the surroundings, typically characterized by aggressive or rule-breaking behaviors [36]. Previous research has indicated that around 25% of preschool children in Taiwan score within the clinical range for total problems [37]. The prevalence of internalizing syndromes is approximately twice that of externalizing syndromes (25.1% vs. 11.2%). The three most commonly reported syndromes include physical discomfort (14.9%), withdrawal (13.5%), and sleep problems (8.5%) [37]. Our findings align with these previous observations; in our study, we also noted a higher prevalence of internalizing syndromes compared to externalizing syndromes among preschool children in Taiwan.

During embryonic development, testosterone and estradiol play crucial roles in brain sexual differentiation. Estradiol and testosterone directly affect the developing neural circuits in the embryo. These hormones are responsible for the masculinization of the brain, while estradiol is involved in postnatal sexual development and the feminization of females. Endocrine-disrupting chemicals may impact brain development by interfering with the function of sex hormones [38].

Previous studies have shown that perinatal exposure to DEHP increases depressive-like and anxiety-like behaviors in mice. Maternal exposure to BPA has been associated with a greater likelihood of anxiety, depressive symptoms, and memory impairment in male offspring [39]. Additionally, higher urinary BPA concentrations have been linked to ADHD in American children, with a stronger association in boys [40]. However, in our study, there was no significant difference in BPA levels, suggesting that the higher prevalence of ADHD symptoms in boys compared to girls may not be attributed to BPA exposure. Nevertheless, we observed that postnatal urinary MBzP levels were higher in boys than in girls. Previous literature has indicated that, in boys, higher maternal concentrations of high-molecular-weight phthalates (MBzP, ∑DiNP, and ∑DEHP) are nearly significantly correlated with an increase in ADHD symptom scores [41]. In our study, we did not find prenatal differences; instead, the postnatal MBzP levels were higher. Daniel et al. (2020) examined sex-specific associations between phthalate exposure at ages three and five and ADHD-related behaviors at age seven. They found that at age three, higher urinary MBzP concentrations were associated with more ADHD symptoms in girls [18]. It is possible that ADHD is influenced by multiple factors that vary according to different eras and living environments.

We observed that the concentration of MBzP in the urine of preschool boys was higher than that in preschool girls. Previous studies have also reported higher urinary concentrations of PMBP and PDEHPm or MBzP in boys compared to girls [42,43]. This difference between sexes may be related to the variation in consumer products used by boys and girls, or differences in metabolic capabilities. However, the higher concentration of MBzP in preschool boys’ urine should not be interpreted as a contributing factor to the higher prevalence of ADHD among them, as there is no correlation between the ADHD T-scores in young children and urinary MBzP concentrations. In this study, we found that the concentrations of MEP and MnBP in mothers were higher compared to those in children. This difference may be due to varying sources and pathways of phthalate exposure between young children and adults [23]. Literature reviews indicate that, overall, the concentrations of phthalate metabolites tend to decrease with age [42]; however, different types of phthalates may still be associated with variations in lifestyle habits across different regions. The elevated MEP levels in pregnant women compared to young children could be associated with the use of cosmetics during pregnancy [44,45,46].

MEP is a metabolite of di(2-ethylhexyl) phthalate (DEHP), while MnBP is a metabolite of mono-n-butyl phthalate (MBP), and MBP itself is also a metabolite of di-n-butyl phthalate (DBP). MEP is commonly found in items such as toothbrushes, toys, food packaging, cosmetics, pesticides, and aspirin, whereas MnBP is commonly used in backing for carpets, paints, adhesives, insecticides, hair sprays, and nail polishes [47,48].

In 2021, a study explored the associations of phthalate metabolites in the urine of 754 Black women in Detroit, Michigan. It found that the correlations among low molecular weight phthalates were relatively strong, as were the correlations among high molecular weight phthalates [46]. However, the correlation between high molecular weight and low molecular weight phthalates was lower. Our research revealed a very high correlation among different phthalate metabolites in mothers. Specifically, the correlation among low molecular weight phthalates (such as MEP and MnBP) was strong, while the correlation with high molecular weight phthalates (such as MBzP and MEHP) was weaker, and vice versa. BPA exhibited some correlation with other phthalates as well. Previous reports indicated that the correlations of DEHP metabolites (MEHP, MEHHP, MEOHP, and MECPP) between maternal and child samples were weak and negative [45]. However, our study demonstrated that there was no significant correlation between the concentrations of urinary phthalate metabolites in mothers and their children.

Environmental factors during prenatal and early childhood development may negatively impact children’s neurodevelopment at critical moments by altering metabolic pathways and physiological systems. Thyroid imbalances in pregnant women may lead to permanent neurodevelopmental consequences in children, including attention deficits, hyperactivity, and autism spectrum disorders, as well as cognitive and behavioral dysfunctions. Disruption of sex hormone functions may also have dimorphic effects on brain development [14]. Thus, exposure to environmental hormones during the prenatal and early childhood development stages may play a significant role in the later development of diseases. We found that distinguishing by symptoms that reach clinical significance, an increased urinary MnBP concentration in pregnant women during the second trimester was associated with a higher risk of total problems (OR = 19.32, 95% CI: 1.80–43.129, *p* = 0.04), anxiety (OR = 33.58, 95% CI: 2.16–521.18, *p* = 0.01), and sleep issues (OR = 41.34, 95% CI: 1.04–1632.84, *p* = 0.04) in infants at 1.5 to 3 years of age. For infants, a higher urinary MnBP concentration was also associated with an increased risk of total problems (OR = 7.06, 95% CI: 1.01–49.05, *p* = 0.04) and internalizing problems (OR = 11.04, 95% CI: 1.27–95.72, *p* = 0.01). Given that MnBP is a metabolite of MBP and DBP, these results suggest that prenatal and postnatal exposure to DBP has significantly different impacts on the emotional and behavioral development of children.

In a 2015 study in Taiwan, urine samples were collected from 122 mothers during the third trimester of pregnancy. After adjusting for the child’s sex, intelligence, and family income, it was found that MBP levels had a significant correlation with externalizing behavior scores and criminal behavior ratings in 8-year-old children [27].

A recent study indicated that urinary samples of MnBP and Σ diethyl phthalate in pregnant women are linked to poorer social awareness in newborns [17]. Compared to previous studies, our research indicates that the impact of phthalates on children’s behavioral development may begin as early as the second trimester. Furthermore, the effects of prenatal and postnatal exposure to the same phthalate differ significantly in terms of child behavior development. Previous studies primarily focused on the direct correlation between phthalate concentrations and the T-score of behavioral problems. In contrast, our research further distinguishes between clinical and non-clinical significance to explore the effects of phthalates on children’s behavioral development. This differentiation not only aids in more accurately assessing the potential risks of phthalates but also provides clinically meaningful insights that can help in developing targeted intervention measures.

Our study has several limitations. Since we focused on early childhood behavioral development, we did not adjust for intelligence data. Additionally, the limited number of cases meant that fewer children reached the clinical range on the CBCL. Furthermore, we do not know the sources of phthalate exposure for the cases, nor the potential effects of other unmeasured chemicals in the environment on the results.

## 5. Conclusions

Our research found that maternal urinary MnBP concentration during the second trimester of pregnancy is correlated with the total problems, anxiety, and sleep issues in children aged 1.5 to 3 years, resulting in scores that reach clinically significant levels on the CBCL. Additionally, the urinary MnBP concentration in infants postnatally is also correlated with the total problems and internalizing issues in children aged 1.5 to 3 years, leading to scores that reach clinically significant levels. Because MEP is a metabolite of DBP, prenatal and postnatal exposure to DBP has varying negative impacts on children’s behavioral development.

## 6. Limitations

EDCs include a wide range of substances, such as bisphenol A (BPA) and phthalates. Other common EDCs are industrial chemicals like dioxins and dioxin-like polychlorinated biphenyls (PCBs); pesticides such as dichlorodiphenyltrichloroethane (p,p′-DDT) and its metabolite dichlorodiphenyldichloroethylene (p,p′-DDE), as well as hexachlorobenzene (HCB); per- and polyfluoroalkyl substances (PFASs) like perfluorooctane sulfonate (PFOS) and perfluorooctanoic acid (PFOA); brominated flame retardants (BFRs) like polybrominated diphenyl ethers (PBDEs); and polycyclic aromatic hydrocarbons (PAHs) [49].

Exposure to multiple EDCs, often referred to as the “cocktail effect”, can impact the endocrine system [50] and influence neurodevelopment in early childhood [38]. However, our study did not examine the effects of other EDCs. For example, food and beverage packaging can also contain phthalates [51]. Maternal exposure to such packaging may influence the concentration of phthalate metabolites in urine [52]. Although a wide range of consumer products contains phthalates, and the amount entering the body is influenced by various factors, using urinary phthalate metabolites allowed us to assess exposure to specific phthalates. However, we did not examine the effects of other hormonal factors (e.g., maternal estrogen or contraceptive use), which represents a limitation of our research.

## Figures and Tables

**Table 1:** The basic characteristics of the study population.

	All	Male	Female	*p*
Case Number	491	245	246	
Age (months)	40.96 ± 7.71	41.13 ± 7.36	40.78 ± 8.05	0.607
Mother’s characteristics				
Age (years)	33.28 ± 4.43	33.29 ± 4.51	33.27 ± 4.36	0.949
BMI	23.55 ± 4.68	23.24 ± 4.34	23.86 ± 4.99	0.145
Smoking	22 (4.4%)	7 (2.9%)	15 (6.1%)	0.083
Education level				
High School	43 (8.7%)	19 (7.7%)	24 (9.8%)	0.256
College	359 (73.1%)	180(73.5%)	179 (72.8%)	
Graduate School	89 (18.1%)	46 (18.8%)	43 (17.5%)	
Parity				0.859
1	307 (62.5%)	151 (61.6%)	156 (63.4%)	
2	155 (31.6%)	82 (22.5%)	73 (29.7%)	
3	24 (4.9%)	11 (4.5%)	13 (5.3%)	
≧4	5 (1%)	1 (0.4%)	4 (1.6%)	
Father’s characteristics				
Age (year)	35.20 ± 5.56	35.25 ± 5.63	35.15 ± 5.49	0.843
Smoking	38 (8.0%)	20 (8.3%)	18 (7.6%)	0.766
Education level				
High School	78 (15.8%)	32 (13.0%)	46 (18.1%)	0.055
College	317 (64.6%)	162 (66.1%)	155 (63.0%)	
Graduate School	96 (19.5%)	51 (20.8%)	45 (18.3%)	
Either parent smoking	55 (11.2%)	25 (10.2%)	30 (12.2%)	0.444

BMI, body mass index; Mean ± SD or N (%) median IQR (median difference).

**Table 2:** The CBCL scores in the total study population and different sexes.

T Score	All N = 491	Male N = 245	Female N = 246	*p*
Broadband scales				
Total problems	52.05 ± 10.87	52.52 ± 9.94	51.56 ± 11.72	0.318
Internalizing	54.33 ± 11.04	54.31 ± 10.40	54.35 ± 11.65	0.965
Externalizing	47.75± 9.59	48.52 ± 8.66	46.99 ± 10.40	0.077
Syndrome scales				
Emotionally reactive	56.62 ± 7.19	56.52 ± 6.39	56.72 ± 7.91	0.757
Anxious/depressed	54.44 ± 6.13	54.31 ± 5.08	54.57 ± 7.03	0.641
Somatic complaints	58.18 ± 8.64	57.91 ± 8.19	58.45 ± 9.07	0.492
Withdrawn	58.03 ± 8.29	57.95 ± 7.97	58.12 ± 8.62	0.824
Sleep problems	55.30 ± 7.20	55.36 ± 6.91	55.23 ± 7.48	0.835
Attention problems	53.48 ± 4.92	53.89 ± 4.85	53.08 ± 4.97	0.066
Aggressive behavior	52.79 ± 5.71	52.82 ± 4.65	52.76 ± 6.60	0.907
DSM-oriented scales				
Affective problems	58.20 ± 8.47	58.05 ± 7.85	58.35 ± 9.06	0.691
Anxiety problems	55.63 ± 7.44	55.32 ± 6.63	55.94 ± 8.17	0.356
PDP	57.24 ± 8.30	57.41 ± 7.73	57.07 ± 8.85	0.655
ADHD problems	53.26 ± 5.36	53.90 ± 5.87	52.62 ± 4.72	0.008 *
ODD problems	52.46 ± 4.84	52.50 ± 4.57	52.42 ± 5.09	0.849

CBCL = Child Behavior Checklist; PDP = pervasive developmental problems; ADHD = attention deficit/hyperactivity; ODD = oppositional/defiant; *p* = male vs. female. * *p* < 0.05

**Table 3:** After correction for urinary creatinine, the metabolites of urine plasticizers of mother and child in the total study population and different sex groups (μg/g-Cr).

Mother Metabolites of Urine Plasticizers	All N = 309	Male N = 155	Female N = 154	*p*
MEP	14.074 ± 8.344 (0.10–48.00)	14.527 ± 8.306 (0.10–37.70)	13.618 ± 8.384 (0.10–48.00)	0.339
MnBP	10.546 ± 5.797 (0.32–34.30)	10.489 ± 5.615 (0.32–25.70)	10.604 ± 5.993 (0.60–34.30)	0.862
MBzP	0.512 ± 0.676 (0.08–6.90)	0.526 ± 0.752 (0.10–6.90)	0.497 ± 0.591 (0.08–4.70)	0.702
MEHP	1.293 ± 1.134 (0.10–7.70)	1.301 ± 1.214 (0.10–7.70)	1.286 ± 1.051 (0.10–6.00)	0.908
BPA	1.132 ± 0.881 (0.10–7.10)	1.168 ± 0.889 (0.10–5.00)	1.095 ± 0.874 (0.10–7.10)	0.466
Infant Metabolites of Urine Plasticizers	All N = 98	Male N = 50	Female N = 48	*p*
MEP	10.726 ± 8.137 (0.20–39.20)	11.442 ± 7.875 (0.20–39.20)	9.981 ± 8.420 (0.20–34.00)	0.377
MpnBP	8.749 ± 12.463 (0.10–116.20)	10.012 ± 16.573 (0.10–116.20)	7.433 ± 5.546 (0.10–22.40)	0.308
MBzP	0.759 ± 1.024 (0.10–4.50)	0.750 ± 1.024 (0.10–4.50)	0.401 ± 0.608 (0.10–3.20)	0.043 *
MEHP	2.035 ± 2.129 (0.10–9.00)	1.759 ± 2.077 (0.10–9.00)	2.322 ± 2.165 (0.10–9.00)	0.192
BPA	1.112 ± 0.833 (0.10–4.00)	0.969 ± 0.660 (0.10–3.00)	1.262 ± 0.965 (0.10–4.00)	0.084

MEP = Mono-ethyl phthalate, MnBP = Mono-n-butyl phthalate, MBzP = Mono-benzyl phthalate, MEHP = Mono-2-ethylhexyl phthalate, BPA = Bisphenol A. * *p* < 0.05.

**Table 4:** Linear regression to identify mother’s (prenatal) metabolite factors that correlated with CBCL scores (adjusted for sex, parents’ age, parents’ smoking status, maternal parity, mother’s BMI, parents’ education level) (coefficient/*p*).

	MEP		MnBP		MBzP		MEHP		BPA	
	Coefficient (95% CI)	(*p*)	Coefficient (95% CI)	(*p*)	Coefficient (95% CI)	(*p*)	Coefficient (95% CI)	(*p*)	Coefficient (95% CI)	(*p*)
Broadband scales										
Total problems	0.11(−0.13–7.14)	0.06	0.11 (−0.22–7.89)	0.06	−0.01 (−4.18–3.39)	0.83	−0.05 (−5.60–1.93)	0.33	0.07 (−1.43–5.68)	0.24
Internalizing	0.04 (−2.17–5.00)	0.43	0.03 (−2.97–5.01)	0.61	−0.65 (−5.77–1.62)	0.27	−0.04 (−4.95–2.42)	0.50	0.03 (−2.33–4.638)	0.51
Externalizing	0.10 (−0.47–6.07)	0.09	0.15 (1.24–8.49)	0.01	0.03 (−2.26–4.54)	0.50	−0.02 (−3.97–2.81)	0.73	0.06 (−1.36–5.03)	0.25
Syndrome scales										
Emotionally reactive	0.05 (−1.30–3.33)	0.39	0.05 (−1.31–3.84)	0.33	0.04(−1.57–3.21)	0.50	−0.04 (3.36–1.40)	0.42	0.10 (0.29–4.19)	0.08
Anxious/depressed	0.02 (−1.67–2.28)	0.76	**0.12** **(0.08–4.44)**	0.04 *	0.01 (−1.88–2.19)	0.88	−0.05 (−2.91–1.13)	0.38	−0.01 (−1.95–1.88)	0.97
Somatic complaints	0.01 (−2.57–3.33)	0.80	−0.02 (3.99–2.56)	0.66	**−0.15** **(−6.99–−0.97)**	0.01 *	−0.02 ( −2.35–3.71)	0.65	0.01 (−2.54–3.19)	0.82
Withdrawn	0.11 (−0.11–5.20)	0.06	0.02 (−2.40–3.54)	0.70	−0.07 (−4.59–0.91)	0.19	−0.02 (−3.25–2.24)	0.71	−0.02 (−3.11–2.08)	0.69
Sleep problems	0.11 (−0.08–4.61)	0.06	0.10 (−0.30–4.92)	0.08	0.04 (−1.55–3.31)	0.47	−0.02 (−3.04–1.81)	0.61	0.02 (−1.86–2.72)	0.71
Attention problems	0.10 (0.17–3.30)	0.07	0.10 (−0.14–3.71)	0.07	−0.01 (−1.85–1.75)	0.95	−0.05 (−2.63–0.95)	0.35	0.04 (−1.13–2.25)	0.51
Aggressive behavior	0.06 (−0.75–2.82)	0.25	**0.15** **(0.67–4.61)**	0.01 *	**0.12** **(0.19–3.86)**	0.03 *	−0.01 (−1.88–1.80)	0.96	0.02 (−1.35–2.13)	0.65
DSM-oriented scales										
Affective problems	0.05 (−1.52–4.29)	0.34	0.01 (−2.86–3.61)	0.81	−0.07 (−4.98–1.00)	0.19	−0.21 (−3.53–2.45)	0.72	0.01 (−2.45–3.20)	0.79
Anxiety problems	0.06 (−1.11–3.72)	0.28	0.21 (−0.98–4.38)	0.07	0.04 (−1.58–3.40)	0.47	−0.08 (−4.21–0.74)	0.16	0.03 (−1.72–2.97)	0.60
PDP	**0.12** **(0.24–5.81)**	0.03 *	**0.14** **(0.71–6.88)**	0.01 *	−0.01 (−3.15–2.63)	0.86	−0.07 (−4.63–1.11)	0.23	0.03 (−1.95–3.49)	0.57
ADHD problems	**0.13** **(0.37–4.00)**	0.01 *	**0.18** **(1.20–5.21)**	0.01 *	0.10 (−0.09–3.66)	0.06	−0.05 (−2.77–0.98)	0.34	0.07 (−0.65–2.89)	0.21
ODD problems	0.10 (−0.19–2.92)	0.08	**0.16** **(0.63–4.06)**	0.01 *	0.09 (−0.34–2.86)	0.12	0.04 (−1.04–2.17)	0.49	−0.01 (−1.61–1.42)	0.89

CBCL = child behavior checklist, PDP = pervasive development problems, ADHD = attention deficit hyperactive disorder, ODD = oppositional defiant disorder, * *p* < 0.05.

**Table 5:** Multivariable logistic regression to identify mother’s (prenatal) metabolites that favor having clinical problems (adjusted for sex, parents’ age, parents’ smoking status, maternal parity, parents’ education level, mother’s BMI).

	MEP		MnBP		MBzP		MEHP		BPA	
	OR (95% CI)	(*p*)	OR (95% CI)	(*p*)	OR (95% CI)	(*p*)	OR (95% CI)	(*p*)	OR (95% CI)	(*p*)
Broadband scales										
Total problems	1.83 (0.54–6.25)	0.33	**8.81 (1.80–43.12)**	0.01 *	1.17 (0.39–3.46)	0.77	0.41 (0.13–1.27)	0.12	1.67 (0.57–4.90)	0.34
Internalizing	1.33 (0.49–3.58)	0.57	1.66 (0.55–5.03)	0.36	0.69 (0.25–1.86)	0.46	0.91 (0.35–2.37)	0.86	1.67 (0.66–4.21)	0.27
Externalizing	3.47 (0.24–48.58)	0.35	10.25 (0.47–220.65)	0.13	2.69 (0.41–17.55)	0.30	2.09 (0.26–16.33)	0.48	1.49 (0.20–10.90)	0.69
Syndrome scales										
Emotionally reactive	1.66 (0.19–14.22)	0.64	3.84 (0.25–57.44)	0.32	3.02 (0.52–17.38)	0.21	0.49 (0.06–3.84)	0.49	1.01 (0.14–7.08)	0.98
Anxious/depressed	3.65 (0.06–208.23)	0.53	24.34 (0.14–4094.18)	0.22	1.79 (0.08–40.51)	0.71	0.32 (0.01–10.57)	0.52	0.58 (0.02–14.46)	0.74
Somatic complaints	1.31 (0.42–4.09)	0.63	0.59 (0.19–1.77)	0.35	0.33 (0.09–1.16)	0.08	1.44 (0.48–4.33)	0.51	1.03 (0.36–2.92)	0.95
Withdrawn	1.39 (0.34–5.57)	0.63	1.09 (0.25–4.75)	0.89	0.33 (0.07–1.51)	0.15	0.91 (0.24–3.40)	0.89	1.06 (0.31–3.68)	0.91
Sleep problems	8.79 (0.42–183.11)	0.16	**41.34 (1.04–1632.84)**	0.04 *	1.46 (0.16–12.68)	0.72	0.50 (0.06–4.15)	0.52	0.56 (0.07–4.22)	0.57
Attention problems	14.65 (0.06–3349.96)	0.33	178.11 (0.07–451,307.07)	0.19	7.96 (0.10–615.41)	0.34	2.41 (0.03–234.77)	0.70	0.57 (0.01–23.04)	0.77
Aggressive behavior	0.01 (−)	0.99	0.01 (−)	0.99	88,176.56 (−)	0.99	0.01 (−)	0.99	0.01 (−)	0.99
DSM-oriented scales										
Affective problems	1.17 (0.41–0.32)	0.76	0.07 (0.45–4.50)	0.17	0.57 (0.19–1.68)	0.31	0.74 (0.26–2.05)	0.56	1.14 (0.43–2.99)	0.78
Anxiety problems	4.67 (0.52–41.28)	0.16	**33.58 (2.16–521.18)**	0.01 *	3.83 (0.88–16.58)	0.07	0.50 (0.09–2.80)	0.43	1.61 (0.31–8.45)	0.56
PDP	1.90 (0.45–8.01)	0.38	1.68 (0.37–7.62)	0.50	0.72 (0.19–2.68)	0.62	0.29 (0.08–1.08)	0.06	0.84 (0.25–2.77)	0.78
ADHD problems	37.12 (0.60–2275.42)	0.85	13.8 (0.19–998.39)	0.22	0.27 (0.01–4.55)	0.13	0.26 (0.01–4.18)	0.34	1.02 (0.07–14.06)	0.98
ODD problems	1.22 (0.03–39.91)	0.91	29.98 (0.15–5919.40)	0.20	7.90 (0.52–118.79)	0.13	0.98 (0.03–24.84)	0.99	0.70 (0.03–15.96)	0.82

CBCL = child behavior checklist, PDP = pervasive development problems, ADHD = attention deficit hyperactive disorder, ODD = oppositional defiant disorder, * *p* < 0.05.

**Table 6:** Linear regression to identify the children’s (postnatal) metabolites that correlated with CBCL scores (adjusted for sex, parents’ age, parents’ smoking status, maternal parity, parents’ education level, mother’s BMI) (coefficient/*p*).

	MEP		MnBP		MBzP		MEHP		BPA	
	Coefficient (95% CI)	(*p*)	Coefficient (95% CI)	(*p*)	Coefficient (95% CI)	(*p*)	Coefficient (95% CI)	(*p*)	Coefficient (95% CI)	(*p*)
Broadband scales										
Total problems	−0.01 (−4.09–4.04)	0.99	−0.04 (−5.35–3.70)	0.71	0.09 (−3.55–8.14)	0.43	−0.02 (−4.64–3.80)	0.84	−0.07 (−7.68–4.01)	0.53
Internalizing	0.05 (−3.01–5.09)	0.60	−0.02 (−4.97–4.06)	0.84	0.16 (−1.81–9.76)	0.17	−0.16 (−4.51–3.91)	0.88	−0.09 (−8.91–3.46)	0.42
Externalizing	−0.08 (−5.10–2.35)	0.46	−0.10 (−6.08–2.20)	0.35	0.01 (−5.16–5.63)	0.93	−0.10 (−5.62–2.10)	0.36	−0.01 (−5.70–5.08)	0.91
Syndrome scales										
Emotionally reactive	0.02 (−2.30–2.96)	0.80	0.10 (−1.66–4.18)	0.39	0.02 (−3.34–4.26)	0.80	0.05 (−2.03–3.43)	0.61	−0.09 (−5.26–2.30)	0.43
Anxious/depressed	−0.01 (−2.16–2.02)	0.94	−0.06 (−3.01–1.64)	0.56	−0.02 (−3.30–2.75)	0.85	−0.16 (−3.69–0.59)	0.15	−0.09 (−4.26–1.75)	0.41
Somatic complaints	0.07 (1.73–3.64)	0.48	−0.02 (−3.31–2.70)	0.84	0.02 (−3.59–4.19)	0.87	0.02 (−2.52–3.08)	0.84	−0.06 (−5.06–2.69)	0.54
Withdrawn	0.17 (−0.73–5.23)	0.13	**0.27 (0.47–6.99)**	0.02 *	**0.41 (3.18–11.26)**	0.01 *	−0.03 (−3.60–2.66)	0.76	0.04 (−3.49–5.22)	0.69
Sleep problems	0.02 (−2.50–3.10)	0.83	−0.03 (−3.60–2.64)	0.76	0.07 (−2.82–5.24)	0.55	0.10 (−1.53–4.25)	0.35	0.02 (−3.63–4.44)	0.84
Attention problems	0.08 (−1.21–2.78)	0.43	0.01 (−2.21–2.26)	0.98	−0.01 (−2.99–2.80)	0.94	−0.08 (−2.85–1.30)	0.46	0.06 (−2.10–3.68)	0.58
Aggressive behavior	−0.08 (−2.92–1.28)	0.44	−0.01 (−2.51–2.18)	0.88	−0.09 (−4.16–1.91)	0.46	−0.16 (−3.68–0.64)	0.16	0.01 (−2.89–3.19)	0.92
DSM-oriented scales										
Affective problems	0.05 (−2.18–3.55)	0.63	−0.03 (−3.60–2.79)	0.80	0.11 (−2.18–6.05)	0.35	0.03 (−2.51–3.44)	0.75	−0.02 (−4.50–3.77)	0.86
Anxiety problems	0.03 (−2.28–3.21)	0.73	−0.01 (−3.30–2.83)	0.87	0.09 (−2.40–5.51)	0.43	0.01 (−2.82–2.89)	0.97	−0.07 (−5.24–2.67)	0.52
PDP	0.08 (−2.01–4.68)	0.43	0.08 (−2.36–5.10)	0.46	**0.28 (0.91–10.28)**	0.02 *	0.02 (−3.18–3.80)	0.86	0.13 (−1.96–7.64)	0.24
ADHD problems	−0.08 (−3.56–1.53)	0.42	−0.06 (−3.60–2.08)	0.59	−0.01 (−3.89–3.49)	0.91	−0.19 (−4.82–0.38)	0.09	0.14 (−1.28–6.00)	0.20
ODD problems	0.01 (−2.24–2.33)	0.96	0.05 (−2.00–3.09)	0.67	−0.06 (−4.12–2.47)	0.62	−0.09 (−3.32–4.10)	0.42	0.06 (−2.44–4.14)	0.61

CBCL = child behavior checklist, PDP = pervasive development problems, ADHD = attention deficit hyperactive disorder, ODD = oppositional defiant disorder, * *p* < 0.05.

**Table 7:** Multivariable logistic regression to identify the children’s (postnatal) metabolites that favor having clinical problems (adjusted for sex, parents’ age, parents’ smoking status, maternal parity, mother’s BMI).

	MEP		MnBP		MBzP		MEHP		BPA	
	OR (95% CI)	(*p*)	OR (95% CI)	(*p*)	OR (95% CI)	(*p*)	OR (95% CI)	(*p*)	OR (95% CI)	(*p*)
Broadband scales										
Total problems	1.82 (0.46–7.25)	0.39	**7.06 (1.01–49.05)**	**0.04** *	1.92 (0.43–8.55)	0.39	1.12 (0.33–3.76)	0.84	0.94 (0.18–4.83)	0.94
Internalizing	2.09 (0.48–9.00)	0.32	**11.04 (1.27–95.72)**	**0.02** *	1.69 (0.37–7.78)	0.49	0.92 (0.27–3.07)	0.89	0.64 (0.12–2.97)	0.54
Externalizing	0.58 (0.12–2.65)	0.48	0.51 (0.08–3.02)	0.46	**0.01 (0.01–0.46)**	**0.02** *	**0.10 (0.01–0.94)**	**0.04** *	0.56 (0.04–7.28)	0.66
Syndrome scales										
Emotionally reactive	1.52 (0.10–22.72)	0.75	1.29 (0.08–20.14)	0.85	0.37 (0.01–17.48)	0.61	4.56 (0.22–92.25)	0.32	1.33 (0.05–33.47)	0.86
Anxious/depressed	0.62 (0.01–134.58)	0.86	−		0.01 (−)	0.98	0.01 (−)	0.98	0.04 (0.01–134.55)	0.44
Somatic complaints	0.70 (0.10–4.90)	0.72	2.18 (0.10–46.50)	0.61	0.74 (0.02–24.30)	0.87	1.44 (0.98–21.21)	0.78	0.01 (0.01–1.96)	0.08
Withdrawn	6.02 (0.13–277.55)	0.35	9.18 (0.28–369.47)	0.24	0.74 (0.03–14.69)	0.84	0.88 (0.11–6.60)	0.90	1.05(0.06–18.50)	0.97
Sleep problems	4.27 (0.08–214.28)	0.46	0.88 (0.11–6.63)	0.90	1.85 (0.12–28.04)	0.65	1.82 (0.18–17.61)	0.60	2.18 (0.12–36.93)	0.58
Attention problems	−		−		−		−		−	
Aggressive behavior	0.01 (−)	0.99	0.01 (−)	0.99	0.01 (−)	0.99	0.01 (−)	0.99	0.01 (−)	0.99
DSM-oriented scales										
Affective problems	1.24 (0.31–4.95)	0.75	1.07 (0.22–5.06)	0.92	1.16 (0.15–8.81)	0.88	0.96 (0.24–3.86)	0.99	0.45 (0.06–3.42)	0.44
Anxiety problems	2.61 (0.17–38.34)	0.48	9.98 (0.22–435.84)	0.23	0.49 (0.02–8.60)	0.63	0.66 (0.09–4.82)	0.99	0.72 (0.04–11.49)	0.81
PDP	1.25 (0.31–4.92)	0.75	3.86 (0.51–29.08)	0.18	2.04 (0.30–13.80)	0.46	1.06 (0.28–4.00)	0.99	1.98 (0.29–13.36)	0.48
ADHD problems	1.95 (0.12–31.51)	0.63	0.53 (0.07–3.92)	0.53	1.37 (0.05–33.22)	0.84	1.12 (0.11–10.41)	0.99	9.28 (0.19–452.00)	0.26
ODD problems	444.45 (0.001–43,737,926.71)	0.45	19.42 (0.01–69,779.40)	0.47	0.01 (−)	0.98	0.01 (−)	0.99	0.14 (0.01–45.49)	0.50

CBCL = child behavior checklist, PDP = pervasive development problems, ADHD = attention deficit hyperactive disorder, ODD = oppositional defiant disorder, * *p* < 0.05.

## Data Availability

Data are available by asking the corresponding author after publication.

## Supplementary Materials

The following supporting information can be downloaded at: https://www.mdpi.com/article/10.3390/toxics12110795/s1, Figure S1: Differences in paired mother (prenatal) and infant (postnatal) metabolite levels. ** *p* < 0.01. (MEP = Mono-ethyl phthalate, MnBP = Mono-n-butyl phthalate, MBzP = Mono-benzyl phthalate, MEHP = Mono-2-ethylhexyl phthalate, BPA = Bisphenol A); Table S1: The CBCL score in clinical category of total study population and different sex groups; Table S2: Comparison of maternal urinary phthalate metabolites and BPA levels with global studies [52,53,54,55]; Table S3: Stratified analysis for sensitivity analysis (maternal education above high school) and multivariable logistic regression to identify the mother’s (postnatal) phthalate metabolites that affect CBCL scores (adjusted for sex, parents’ age, parents’ smoking status, maternal parity, mother’s BMI) (coefficient/*p*); Table S4: Stratified analysis for sensitivity analysis (maternal education above high school) and multivariable logistic regression to identify the infant’s (postnatal) phthalate metabolites that affect CBCL scores (adjusted for sex, parents’ age, parents’ smoking status, maternal parity, mother’s BMI) (coefficient/*p*); Table S5: Stratified analysis for sensitivity analysis (maternal parity ≤ 1) and multivariable logistic regression to identify the mother’s (postnatal) metabolites that affect CBCL scores (adjusted for sex, parents’ age, parents’ smoking status, parents’ education level, mother’s BMI) (coefficient/*p*); Table S6: Stratified analysis for sensitivity analysis (maternal parity ≤ 1) and multivariable logistic regression to identify the infant’s (postnatal) metabolites that affect CBCL scores (adjusted for sex, parents’ age, parents’ smoking status, parents’ education level, mother’s BMI) (coefficient/*p*); Table S7: Multivariable logistic regression to identify mother’s (prenatal) phthalate metabolites favors having clinical problems (unadjusted for sex, parents’ age, parents’ smoking status, maternal parity, parents’ education level, mother’s BMI) (OR/P); Table S8: Multivariable logistic regression to identify the infant’s (postnatal) phthalate metabolites favors having clinical problems (unadjusted for sex, parents’ age, parents’ smoking status, maternal parity, parents’ education level, mother’s BMI) (OR/P).
