# Supplementary material for: Distinct Impacts of Prenatal and Postnatal Phthalate Exposure on Behavioral and Emotional Development in Children Aged 1.5 to 3 Years
Source: Toxics. 2024 Oct 31;12(11):795. doi: 10.3390/toxics12110795 (PMC11598217; doi:10.3390/toxics12110795)
Supplement: Supplementary file 1 [file toxics-12-00795-s001.zip › toxics-3242962-Supplementary Tables.pdf]

Supplementary Materials

Supplement Table S1. The CBCL score in clinical category of total study population and different gender group.

	Total (N=491)			Male (N=245)			Female (N=246)			*P*
CBCL/1.5 – 5	Normal	Subclinical	Clinical	Normal	Subclinical	Clinical	Normal	Subclinical	Clinical	
Broadband scales										
Total problem	392(79.8%)	32 (6.5%)	67 (13.6%)	192(78.4%)	20 (8.2%)	33 (13.5%)	200(81.3%)	12 (4.9%)	34 (13.8%)	0.337
Internalizing	328(66.8%)	69 (14.1%)	94 (19.1%)	163(66.5%)	33 (13.5%)	49 (20.0%)	165(67.1%)	36 (14.6%)	45 (18.3%)	0.858
Externalizing	445(90.6%)	26 (5.3%)	20 (4.1%)	219(89.4%)	17 (6.9%)	9 (3.7%)	226(91.9%)	9 (3.7%)	11 (4.5%)	0.250
Syndrome scales										
Emotional reactive	410(83.5%)	65 (13.2%)	16 (3.3%)	205(83.3%)	32 (13.1%)	8 (3.3%)	205(83.3%)	33 (13.4%)	8 (3.3%)	0.939
Anxious/depressed	464(94.5%)	12 (2.4%)	15 (3.1%)	230(93.9%)	9 (3.7%)	6 (2.4%)	234(95.1%)	3 (1.2%)	9 (3.7%)	0.992
Somatic complaint	359(73.1%)	64 (13.0%)	68 (13.8%)	179 (73.1%)	31 (12.7%)	34 (13.9%)	179(72.8%)	33 (13.4%)	34 (13.8%)	0.921
Withdrawn	406(82.7%)	37 (7.5%)	48 (9.8%)	202(82.4%)	18 (7.3%)	25 (10.2%)	204(82.9%)	19 (7.7%)	23 (9.3%)	0.814
Sleep problem	458(93.3%)	17 (3.5%)	16 (3.3%)	226(92.2%)	12 (4.9%)	7 (2.9%)	232(94.3%)	5 (2.0%)	9 (3.7%)	0.723
Attention problem	468(95.3%)	15(3.1%)	8 (1.6%)	232(94.7%)	10 (4.1%)	3 (1.2%)	236(95.9%)	5 (2.0%)	5 (2.0%)	0.874
Aggressive behavior	475(96.7%)	9 (1.8%)	7 (1.4%)	237(96.7%)	5 (2.0%)	3 (1.2%)	238(96.7%)	4 (2.0%)	4 (2.0%)	0.874
DSM-oriented scales										
Affective problem	392(79.8%)	29 (5.9%)	70 (14.3%)	201(82.0%)	11 (4.4%)	33 (13.5%)	191(77.6%)	18 (7.3%)	37 (15.0%)	0.355
Anxiety problem	442(90.0%)	23 (4.7%)	26 (5.3%)	218(89.0%)	15 (6.1%)	12 (4.9%)	224(91.1%)	8 (3.73)	14 (5.7%)	0.770
PDP	397(80.9%)	45 (9.2%)	49 (10.0%)	198(80.8%)	22 (9.0%)	25 (10.2%)	199(80.9%)	23 (9.3%)	24 (9.8%)	0.927
ADHD	464(94.5%)	16 (3.3%)	11 (2.2%)	226(92.2%)	12 (4.9%)	7 (2.9%)	238(96.7%)	4 (1.6%)	4 (1.6%)	0.063
ODD problems	478(97.4%)	3(0.6%)	10 (2.0%)	239(97.6%)	2 (0.8%)	4 (1.6%)	239(97.2%)	1 (0.4%)	6 (2.4%)	0.649

CBCL = child behavior checklist, PDP = pervasive developmental problems, ADHD = attention deficit/hyperactivity, ODD = oppositional/defiant, P = male and female P value

Supplement Table S2. Comparison of Maternal Urinary Phthalate Metabolites and BPA Levels with Global Studies

Mothers' metabolites of urine plasticizers	Our case	(Gardener et al., 2022)[52]	(Vilmand et al., 2023) [53]	(Mathew et al., 2021) [54]	(Lu et al., 2024)[55]	
Time	2021-2022	2009-2014	2010 - 2012	2009-2012	2012-2015	
trimester	Second	third	third	Second/ third	third	
Unit	μg/g-Cr	ng/mL-Cr median (min-max)	ng/mL median (min-max)	ng/mg-Cr median (min-max)	μg/g-Cr Median (Q1-Q3)	
Country	Taiwan	U.S.	Denmark	U.S.	Taiwan	
City	Kaohsiung		Odense			
					Male infant	Female infant
Case number	N=309	N=485	N=585	N=323	N = 294	N = 270
MEP	14.074 ± 8.344	67.2 (5.3-5967.0)	18.7 9(ND-5380)	34.12 (2.47-2279.36)	19.41 (7.75-54.77)	19.22 (7.85-55.48)
MnBP	10.546 ± 5.797	9.1 (ND-214.8)	13.45 (ND-239.18)	10.41 (2.34-443.47)	22.31 (13.26-35.7)	22.53 (13.58-35.82)
MBzP	0.512 ± 0.676	7.2 (ND-429.9)	2.44 (ND-71.22)	6.87 (0.47-183.35)	0.60 (0.60 – 1.71)	0.60 (0.60 – 1.42)
MEHP	1.293 ± 1.134	1.2 (ND-38.6)	21.10 (ND-377.74)	2.28 (0.13-75.40)	5.06 (1.89 – 10.21)	4.97 (1.82 – 11.13)
BPA	1.132 ± 0.881	1.6 (ND-214.8)	-	-	-	-

Supplement Table S3. stratified analysis for sensitivity analysis (maternal education above high school)

multivariable logistic regression to identify the mothers' (postnatal) metabolites which affect CBCL scores. (Adjusted for Sex, Parents' age, Parents' smoke status, maternal parity, mother's BMI) (Coefficient/P)

	MEP		MnBP		MBzP		MEHP		BPA	
	OR (95% CI)	(*P*)	OR (95% CI)	(*P*)	OR (95% CI)	(*P*)	OR (95% CI)	(*P*)	OR (95% CI)	(*P*)
Broadband scales										
Total problems	2.121 (0.57-7.80)	0.25	**6.98(1.40-34.69)**	**0.01**	1.24 (0.40-3.78)	0.70	0.36 (0.11-1.14)	0.08	1.79 (0.59-5.43)	0.29
Internalizing	1.70 (0.59-4.89)	0.32	1.85(0.59-5.85)	0.28	0.72 (0.26-1.97)	0.52	0.97 (0.36-2.57)	0.95	1.71 (0.66-4.40)	0.26
Externalizing	2.84 (0.13-59.21)	0.50	4.17(0.18-97.14)	0.37	2.63 (0.25-27.82)	0.42	0.88 (0.07-10.84)	0.92	2.95 (0.24-35.10)	0.39
Syndrome scales										
Emotionally reactive	1.75 (0.18-16.98)	0.62	3.06(0.19-47.73)	0.42	3.33 (0.55-20.42)	0.18	0.39 (0.04-2.06)	0.39	1.63 (0.20-13.19)	0.64
Anxious/depressed	3.36 (0.02-529.10)	0.63	9.99(0.03-2614.46)	0.41	1.19 (0.02-69.42)	0.93	0.03 (0.01-7.01)	0.22	1.03 (0.01-98.71)	0.98
Somatic complaints	1.33 (0.40-4.45)	0.63	0.47(0.19-1.74)	0.32	0.31 (0.08-1.15)	0.08	1.47 (0.47-4.61)	0.50	1.38 (0.46-4.10)	0.56
Withdrawn	1.82 (0.36-9.16)	0.46	1.01(0.22-1.62)	0.99	0.48 (0.10-2.26)	0.35	0.88 (0.21-3.60)	0.86	1.42 (0.37-5.45)	0.60
Sleep problems	24.89 (0.70-876.10)	0.07	**63.94 (1.15-3530.71)**	**0.04**	1.16 (0.12-11.30)	0.89	0.27 (0.02-2.99)	0.29	0.66 (0.07-5.61)	0.70
Attention problems	-		-		-		-		-	
Aggressive behavior	-		-		-		-		-	
DSM-oriented scales										
Affective problems	1.55 (0.496-43.89)	0.44	1.45 (0.45-4.68)	0.52	0.47 (0.15-1.49)	0.20	0.74 (0.25-2.12)	0.57	1.32 (0.48-3.58)	0.57
Anxiety problems	4.891 (0.52-45.99)	0.16	**27.692 (1.83-418.34)**	**0.01**	3.93 (0.86-17.78)	0.07	0.42 (0.07-2.45)	0.34	2.16 (0.38-12.24)	0.38
PDP	1.91 (0.43-8.36)	0.38	1.56 (0.35-6.96)	0.55	0.78 (0.21-2.95)	0.72	0.27 (0.07-1.02)	0.06	1.02 (0.30-3.46)	0.96
ADHD problems	51.47 (0.77-3441.46)	0.06	17.81 (0.24-1298.64)	0.18	0.12 (0.01-4.07)	0.24	0.2 5(0.01-4.16)	0.33	0.99 (0.07-13.79)	0.99
ODD problems	0.42 (0.01-13.53)	0.62	3.41 (0.01-962.25)	0.66	8.61 (0.25-291.06)	0.23	0.06 (0.01-5.60)	0.23	1.31 (0.02-70.18)	0.89

CBCL = child behavior checklist, PDP = pervasive development problems, ADHD = attention deficit hyperactive disorder, ODD = oppositional defiant disorder, * $P < 0.05$

Supplement Table S4. stratified analysis for sensitivity analysis (maternal education above high school)

multivariable logistic regression to identify the infants' (postnatal) metabolites which affect CBCL scores. (Adjusted for Sex, Parents' age, Parents' smoke status, maternal parity, mother's BMI) (Coefficient/P)

	MEP		MnBP		MBzP		MEHP		BPA	
	OR (95% CI)	(*P*)	OR (95% CI)	(*P*)	OR (95% CI)	(*P*)	OR (95% CI)	(*P*)	OR (95% CI)	(*P*)
Broadband scales										
Total problems	2.74(0.50-15.02)	0.24	**15.59(1.48-163.53)**	**0.02**	**4.24(0.79-22.57)**	**0.09**	1.33(0.37-4.69)	0.65	1.21(0.22-6.64)	0.82
Internalizing	2.97(0.52-16.74)	0.21	**16.11(1.47-176.65)**	**0.02**	3.11(0.57-16.81)	0.18	0.93(0.27-3.22)	0.91	0.70(0.14-3.47)	0.66
Externalizing	0.49(0.08-2.93)	0.43	0.63(0.09-4.01)	0.62	0.03(0.01-3.51)	0.15	0.17(0.01-1.51)	0.11	1.09(0.06-17.29)	0.94
Syndrome scales										
Emotionally reactive	1.55(0.11-21.63)	0.74	1.28(0.09-17.40)	0.84	-0.76(0.02-21.44)	0.87	4.14(0.24-70.92)	0.32	1.21(0.06-24.65)	0.89
Anxious/depressed	-		-		-		-		-	
Somatic complaints	1.09(0.11-10.29)	0.93	2.23(0.10-48.64)	0.61	2.50(0.05-126.29)	0.64	1.86(0.13-25.19)	0.63	0.05(0.01-1.98)	0.11
Withdrawn	5.14(0.14-187.19)	0.37	6.50(0.26-159.43)	0.25	1.12(0.06-19.68)	0.93	0.92(0.12-6.87)	0.93	1.04(0.06-16.24)	0.97
Sleep problems	4.14(0.13-123.37)	0.41	0.84(0.11-6.08)	0.86	1.90(0.12-28.64)	0.89	2.33(0.25-21.09)	0.45	2.47(0.15-38.49)	0.51
Attention problems	4.14(0.13-123.37)	0.41	-		-		-		-	
Aggressive behavior	-		-		-		-		-	
DSM-oriented scales										
Affective problems	1.66(0.32-8.45)	0.53	1.20(0.24-5.89)	0.82	4.16(0.40-43.23)	0.22	0.94(0.21-4.18)	0.94	0.50(0.06-4.14)	0.52
Anxiety problems	5.14(0.14-187.19)	0.37	6.50(0.26-159.43)	0.25	1.12(0.06-19.68)	0.93	0.92(0.12-6.87)	0.93	0.97(0.06-16.24)	1.04
PDP	1.25(0.32-4.91)	0.74	3.43(0.51-22.93)	0.20	2.39(0.38-14.90)	0.35	1.09(0.29-4.05)	0.89	1.98(0.30-12.74)	0.47
ADHD problems	2.60(0.18-36.39)	0.47	0.50(0.06-3.83)	0.55	0.88(0.39-20.00)	0.94	1.24(0.14-10.70)	0.84	8.97(0.18-440.38)	0.26
ODD problems	-		-		-		-		-	

CBCL = child behavior checklist, PDP = pervasive development problems, ADHD = attention deficit hyperactive disorder, ODD = oppositional defiant disorder, * *P* <0.05

Supplement Table S5. stratified analysis for sensitivity analysis (maternal parity ≤1)

multivariable logistic regression to identify the mother (postnatal) metabolites which affect CBCL scores. (Adjusted for Sex, Parents' age, Parents' smoke status, Parents' education level, mother's BMI) (Coefficient/P)

	MEP		MnBP		MBzP		MEHP		BPA	
	OR (95% CI)	(*P*)	OR (95% CI)	(*P*)	OR (95% CI)	(*P*)	OR (95% CI)	(*P*)	OR (95% CI)	(*P*)
Broadband scales										
Total problems	2.17 (0.58-8.05)	0.24	**8.05 (1.63-39.54)**	**0.01**	1.26(0.42-3.73)	0.67	0.43(0.13-1.35)	0.15	1.61 (0.54-0.47)	0.38
Internalizing	1.54 (0.53-4.47)	0.42	1.75 (0.55-5.50)	0.33	0.84(0.31-2.29)	0.74	0.96(0.35-2.58)	0.93	1.54 (0.60-3.96)	0.36
Externalizing	3.48 (0.23-51.53)	0.36	12.35 (0.56-268.77)	0.11	2.56(0.38-16.95)	0.32	2.33 (0.28-19.40)	0.43	1.50 (0.20-11.22)	0.68
Syndrome scales										
Emotionally reactive	1.86 (0.16-21.67)	0.61	4.46 (0.27-73.50)	0.29	4.61(0.79-26.82)	0.08	0.31 (0.03-2.85)	0.30	0.87 (0.11-6.91)	0.89
Anxious/depressed	5.10 (0.06-386.44)	0.46	27.92 (0.17-4420.95)	0.19	1.61(0.07-36.52)	0.76	0.39 (0.01-11.66)	0.59	0.58(0.02-15.68)	0.74
Somatic complaints	1.27 (0.40-4.03)	0.68	0.61 (0.20-1.82)	0.38	0.28(0.07-1.01)	0.06	1.39 (0.45-4.29)	0.55	1.04(0.36-2.96)	0.93
Withdrawn	1.98 (0.40-9.65)	0.39	1.06 (0.23-4.78)	0.93	0.46(0.10-2.07)	0.31	0.68 (0.17-2.73)	0.59	1.39(0.38-5.09)	0.61
Sleep problems	8.25(0.40-168.09)	0.17	**37.443 (0.99-1411.70)**	**0.05**	1.38(0.16-12.05)	0.76	0.50 (0.06-4.19)	0.53	0.53(0.07-4.04)	0.54
Attention problems	55.05(0.08-36383.13)	0.22	242.24 (0.09-653377.14)	0.17	12.94(0.12-1318.68)	0.27	2.27 (0.02-184.34)	0.71	1.06(0.01-58.81)	0.97
Aggressive behavior	-		-		-		-		-	
DSM-oriented scales										
Affective problems	1.30(0.43-3.88)	0.63	1.26(0.40-3.92)	0.68	0.63(0.21-1.90)	0.42	0.67 (0.23-1.92)	0.45	1.13 (0.42-3.02)	0.79
Anxiety problems	4.73(0.52-42.97)	0.16	**29.85(**2.00-445.71**)**	**0.01**	3.46(0.79-15.14)	0.09	0.49 (0.08-2.80)	0.42	1.54 (0.29-8.07)	0.60
PDP	2.07(0.46-9.27)	0.34	1.82(0.38-8.50)	0.44	0.77(0.20-2.91)	0.70	**0.18 (0.04-0.72)**	**0.01**	1.09 (0.32-3.71)	0.89
ADHD problems	35.66(0.57-2202.46)	0.08	13.42(0.19-923.17)	0.22	0.13(0.01-4.15)	0.24	0.27 (0.01-4.28)	0.35	0.93 (0.06-12.90)	0.96
ODD problems	1.17(0.32-43.51)	0.92	29.28(0.15-5420.68)	0.20	7.74(0.49-121.40)	0.14	0.96 (0.03-25.77)	0.98	0.65 (0.02-16.49)	0.79

CBCL = child behavior checklist, PDP = pervasive development problems, ADHD = attention deficit hyperactive disorder, ODD = oppositional defiant disorder, * *P* <0.05

Supplement Table S6. stratified analysis for sensitivity analysis (maternal parity ≤1)

multivariable logistic regression to identify the infants' (postnatal) metabolites which affect CBCL scores. (Adjusted for Sex, Parents' age, Parents' smoke status, Parents' education level, mother's BMI) (Coefficient/P)

	MEP		MnBP		MBzP		MEHP		BPA	
	OR(95% CI)	(*P*)	OR(95% CI)	(*P*)	OR(95% CI)	(*P*)	OR(95% CI)	(*P*)	OR(95% CI)	(*P*)
Broadband scales										
Total problems	1.95(0.50-7.54)	0.33	**7.26(1.04-50.79)**	**0.04**	2.05(0.46-9.07)	0.34	1.09(0.33-3.61)	0.88	0.92(0.18-4.70)	0.92
Internalizing	2.20(0.53-9.04)	0.27	**11.60(1.34-100.42)**	**0.02**	1.83(0.41-8.23)	0.42	0.92(0.28-3.01)	0.89	0.63(0.13-3.03)	0.56
Externalizing	0.58(0.12-2.63)	0.48	0.49(0.08-2.76)	0.42	**0.01(0.01-0.49)**	**0.02**	**0.08(0.01-0.80)**	**0.03**	0.58(0.04-7.06)	0.67
Syndrome scales										
Emotionally reactive	1.54(0.10-22.96)	0.75	1.25(0.08-18.79)	0.86	0.40(0.09-19.04)	0.64	4.40(0.21-88.41)	0.33	1.32(0.05-32.57)	0.86
Anxious/depressed	-		-		-		-		-	
Somatic complaints	0.68(0.10-4.74)	0.70	1.92(0.10-34.38)	0.65	0.73(0.02-24.63)	0.86	1.31(0.08-19.29)	0.84	0.01(0.01-1.82)	0.08
Withdrawn	5.74(0.12-258.20)	0.36	6.24(0.22-176.31)	0.28	0.85(0.04-16.73)	0.91	0.74(0.09-5.79)	0.78	1.00(0.06-16.51)	0.99
Sleep problems	3.30(0.11-93.74)	0.48	0.88(0.11-6.72)	0.91	2.07(0.14-30.62)	0.89	1.83(0.20-16.33)	0.58	2.20(0.13-35.92)	0.57
Attention problems	-		-		-		-		-	
Aggressive behavior	-		-		-		-		-	
DSM-oriented scales										
Affective problems	1.26(0.30-5.15)	0.74	0.99(0.23-4.15)	0.98	1.28(0.17-9.37)	0.80	0.93(0.22-3.95)	0.93	0.57(0.08-3.82)	0.56
Anxiety problems	2.46(0.17-34.98)	0.50	6.71(0.12-212.49)	0.28	0.52(0.03-9.37)	0.66	0.56(0.07-4.22)	0.57	0.69(0.04-10.35)	0.79
PDP	1.26(0.31-5.06)	0.73	3.12(0.46-20.83)	0.23	2.25(0.34-14.72)	0.39	0.94(0.24-3.65)	0.93	1.94(0.29-12.88)	0.49
ADHD problems	2.01(0.12-32.19)	0.61	0.53(0.07-3.84)	0.53	1.46(0.06-35.12)	0.81	1.08(0.11-10.04)	0.94	9.03(0.19-428.44)	0.26
ODD problems	-		-		-		-		-	

CBCL = child behavior checklist, PDP = pervasive development problems, ADHD = attention deficit hyperactive disorder, ODD = oppositional defiant disorder, * *P* <0.05

Supplement Table S7 Multivariable logistic regression to identify mothers' (prenatal) metabolites favors of having clinical problems (Unadjusted for Sex, Parents' age, Parents' smoke status, maternal parity, Parents' education level, mother's BMI). (OR/P)

	MEP		MnBP		MBzP		MEHP		BPA	
	OR (95% CI)	(*P*)	OR (95% CI)	(*P*)	OR (95% CI)	(*P*)	OR (95% CI)	(*P*)	OR (95% CI)	(*P*)
Broadband scales										
Total problems	1.76(0.57-0.43)	0.32	**5.06(1.28-19.95)**	**0.02**	1.40(0.55-3.59)	0.47	0.50(0.18-1.40)	0.19	1.03(0.406-2.64)	0.94
Internalizing	1.36(0.53-3.47)	0.51	1.43(0.51-3.97)	0.48	0.86(0.36-2.07)	0.74	0.88(0.36-2.16)	0.79	1.11(0.48-2.55)	0.80
Externalizing	1.53(0.24-9.71)	0.64	1.98(0.25-15.31)	0.51	3.70(0.92-14.82)	0.06	1.42(0.26-7.57)	0.67	0.44(0.10-1.86)	0.26
Syndrome scales										
Emotionally reactive	1.47(0.19-11.46)	0.71	3.02(0.26-34.67)	0.37	2.18(0.42-11.21)	0.34	0.77(0.11-5.08)	0.79	1.02(0.17-5.87)	0.98
Anxious/depressed	0.77(0.08-7.27)	0.82	2.38(0.10-57.36)	0.59	1.76(0.18-16.63)	0.62	0.57(0.04-7.16)	0.67	0.84(0.08-8.40)	0.88
Somatic complaints	1.21(0.41-3.51)	0.72	0.58(0.20-1.65)	0.30	0.46(0.15-1.40)	0.17	1.27(0.45-3.60)	0.64	1.16(0.43-3.09)	0.75
Withdrawn	1.65(0.41-6.50)	0.47	1.21(0.30-4.86)	0.78	0.39(0.10-1.50)	0.17	0.99(0.28-3.39)	0.98	1.07(0.34-3.41)	0.89
Sleep problems	11.39(0.53-241.40)	0.11	**40.32(1.14-1416.54)**	**0.04**	1.08(0.13-8.78)	0.93	0.59(0.06-5.27)	0.63	0.47(0.07-3.14)	0.44
Attention problems	3.92(0.04-373.19)	0.55	20.64(0.08-4963.16)	0.27	1.94(0.08-43.40)	0.67	2.18(0.06-74.23)	0.66	0.48(0.02-10.23)	0.64
Aggressive behavior	0.41(0.01-23.06)	0.66	5.86(0.01-30849.03)	0.68	1.58(0.01-395.31)	0.87	22,79(0.05-9157.10)	0.30	0.05(0.01-6.22)	0.22
DSM-oriented scales										
Affective problems	1.04(0.39-2.73)	0.93	1.20(0.404-3.60)	0.73	0.53(0.19-1.48)	0.23	0.63(0.24-1.70)	0.37	1.07(0.43-2.67)	0.88
Anxiety problems	2.36(0.38-14.70)	0.35	8.65(0.95-78.89)	0.06	2.64(0.71-9.82)	0.14	0.50(0.11-2.34)	0.38	1.34(0.31-5.79)	0.69
PDP	1.96(0.51-7.45)	0.32	2.03(0.49-2.03)	0.32	0.70(0.22-2.27)	0.56	0.32(0.10-1.06)	0.06	0.90(0.31-2.62)	0.85
ADHD problems	**22.56(0.58-875.33)**	**0.09**	11.78(0.27-497.59)	0.19	0.14(0.01-3.12)	0.21	0.37(0.03-4.59)	0.44	0.90(0.08-9.22)	0.93
ODD problems	0.77(0.06-8.93)	0.83	7.30(0.14-373.47)	0.32	3.38(0.35-32.03)	0.28	1.86(0.12-28.99)	0.65	0.61(0.05-6.96)	0.69

CBCL = child behavior checklist, PDP = pervasive development problems, ADHD = attention deficit hyperactive disorder, ODD = oppositional defiant disorder, * *P* <0.05

Supplement Table S8 Multivariable logistic regression to identify the infants' (postnatal) metabolites favors of having clinical problems. (Unadjusted for Sex, Parents' age, Parents' smoke status, maternal parity, Parents' education level, mother's BMI) (OR/P)

	MEP		MnBP		MBzP		MEHP		BPA	
	OR (95% CI)	(*P*)	OR (95% CI)	(*P*)	OR (95% CI)	(*P*)	OR (95% CI)	(*P*)	OR (95% CI)	(*P*)
Broadband scales										
Total problems	2.12 (0.64-7.02)	0.21	2.41 (0.77-7.50)	0.12	1.32 (0.39-4.45)	0.64	1.41 (0.53-3.69)	0.48	1.32 (0.34-5.06)	0.68
Internalizing	1.99 (0.63-6.23)	0.23	2.13 (0.73-6.34)	0.16	1.43 (0.44-1.43)	0.55	1.39 (0.54-3.58)	0.48	1.18 (0.32-4.33)	0.80
Externalizing	0.86 (0.25-2.93)	0.81	0.58 (0.18-1.82)	0.35	0.16 (0.01-1.61)	0.12	0.49 (0.14-1.70)	0.26	0.84 (0.15-4.79)	0.85
Syndrome scales										
Emotionally reactive	1.89 (0.12-28.55)	0.64	1.42 (0.14-14.36)	0.76	1.01 (0.06-16.52)	0.99	5.03 (0.31-80.59)	0.25	0.83 (0.04-15.05)	0.90
Anxious/depressed	0.81 (0.11-5.86)	0.83	1.46 (0.14-14.87)	0.74	0.02 (0.01-3.30)	0.13	0.37 (0.04-2.95)	0.35	0.84 (0.04-15.04)	0.90
Somatic complaints	0.93 (0.20-4.16)	0.92	0.93 (0.20-4.19)	0.93	0.38 (0.03-4.17)	0.43	1.35 (0.28-6.43)	0.70	0.20 (0.02-1.51)	0.12
Withdrawn	6.74(0.40-113.25)	0.18	4.31 (0.55-33.296	0.16	0.52 (0.05-4.99)	0.57	0.68 (0.15-2.99)	0.61	1.00 (0.12-8.39)	0.99
Sleep problems	4.45(0.27-71.77)	0.29	1.40 (0.23-8.47)	0.71	0.81 (0.08-7.90)	0.86	1.61 (0.28-9.26)	0.58	0.71 (0.07-6.71)	0.76
Attention problems	-		-		-		-		-	
Aggressive behavior	3.65 (0.01-1239.19)	0.66	20.68 (0.20-2095.44)	0.19	**-**		-		0.46 (0.01-52.94)	0.75
DSM-oriented scales										
Affective problems	1.19 (0.33-4.31)	0.78	0.70 (0.22-2.19)	0.55	1.60 (0.35-7.20)	0.53	1.08 (0.32-3.61)	0.89	0.75 (0.14-3.90)	0.73
Anxiety problems	1.71 (0.26-11.04)	0.57	1.64 (0.29-9.06)	0.56	0.52 (0.05-4.99)	0.57	0.65 (0.14-2.85)	0.57	0.79 (0.10-6.35)	0.83
PDP			2.61 (0.69-9.81)	0.15	1.35 (0.34-5.32)	0.66	1.10 (0.37-3.24)	0.85	1.90 (0.38-9.46)	0.42
ADHD problems	2.87(0.18-43.76)	0.44	0.69 (0.11-3.81)	0.67	0.94(0.08-11.08)	0.96	1.31 (0.20-8.69)	0.77	6.78 (0.24-191.09)	0.26
ODD problems			3.00 (0.11-42.84)	0.41	0.02 (0.01-3.30)	0.13	0.29 (0.03-2.40)	0.25	0.36 (0.02-5.74)	0.47

CBCL = child behavior checklist, PDP = pervasive development problems, ADHD = attention deficit hyperactive disorder, ODD = oppositional defiant disorder, * *P* <0.05
