# Supplementary figures and images for: Distinct Impacts of Prenatal and Postnatal Phthalate Exposure on Behavioral and Emotional Development in Children Aged 1.5 to 3 Years
Source: Toxics. 2024 Oct 31;12(11):795. doi: 10.3390/toxics12110795 (PMC11598217; doi:10.3390/toxics12110795)

V4

● Prenatal  
● Postnatal

\*\* p value < 0.01

Log( $\mu\text{g/g-Cr}$ )

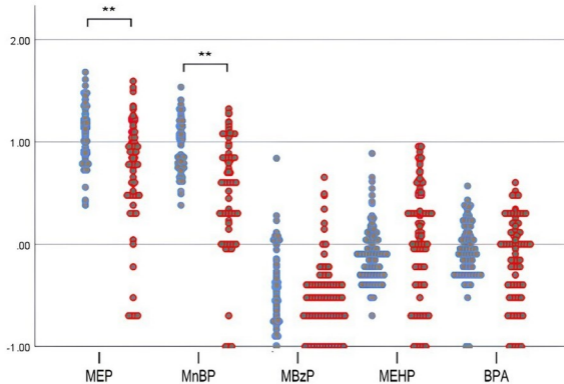

Supplement: Supplementary file 1 [file toxics-12-00795-s001.zip › Supplementary Figure S1.pdf]
